# Buried Stressor Engineering for Position-Controlled InGaAs Quantum Dots with Local Density Variation for Integrated Quantum Photonics

**DOI:** 10.1021/acsphotonics.5c02303

**Published:** 2026-01-07

**Authors:** Martin Podhorský, Maximilian Klonz, Lux Böhmer, Sebastian Kulig, Chirag C. Palekar, Petr Klenovský, Sven Rodt, Stephan Reitzenstein

**Affiliations:** † Institut für Physik Und Astronomie, 26524 Technische Universität Berlin, Hardenbergstraße 36, Berlin D-10623, Germany; ‡ Department of Condensed Matter Physics, Faculty of Science, Masaryk University, Kotlářská 267/2, Brno 61137, Czech Republic; § Czech Metrology Institute, Okružní 31, Brno 63800, Czech Republic

**Keywords:** quantum communication, photonic quantum technologies, site-controlled quantum dots, surface strain engineering, continuum elasticity theory, **k**·**p** method, configuration interaction method

## Abstract

We report on the monolithic, two-step epitaxial growth of site-controlled InGaAs quantum dots via the buried-stressor method with local quantum dot density variation. As a result of high fabrication accuracy, we achieve low lateral displacements of the individual buried-stressor apertures of $17_{-17}^{+19}\mathrm{nm}$ from the mesa centers. We provide extensive microphotoluminescence and cathodoluminescence characterization of the site-controlled quantum dots and give theoretical calculations explaining the effect of the stressor aperture on the quantum dot emission properties, positioning, and density. We show reproducibility of the nucleation process for apertures of the same size and achieve precisely positioned, low- and high-density quantum dot nucleation within one active-layer growth step. The results presented in this work demonstrate the significant potential of the buried-stressor concept in fabricating single photonic chips, simultaneously combining single-photon sources and microlasers featuring different local densities of the site-controlled quantum dots, paving the way for highly functional source modules with applications in photonic quantum technology.

## Graphical abstract

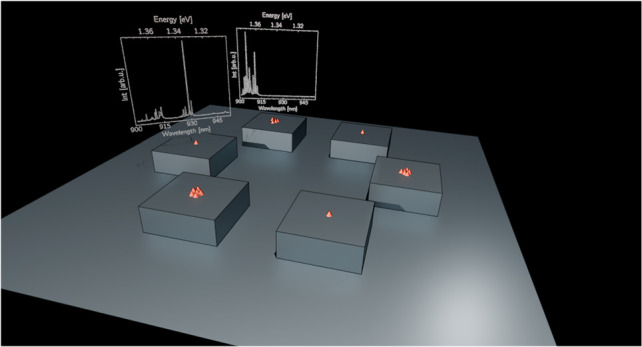

## Introduction

The field of photonic quantum information technology, including quantum computing and quantum communication, requires on-demand single-photon generation via suitable sources. As such, defect centers in solids, quantum emitters in 2D materials, and semiconductor quantum dots (QDs) have proven their significance as single-photon source (SPS) platforms.^1–6^ Among these, self-assembled QDs are of particular interest due to their almost ideal single-photon purity and indistinguishability, combined with a high spontaneous emission rate. These properties enable the highly demanding requirements of advanced quantum optical applications for future photonic quantum technologies to be met, providing a basis for the secure and efficient transmission of quantum information. Furthermore, semiconductor QDs can be employed as the optically active medium in vertically emitting lasers, finding application in areas such as large-capacity optical communication, imaging, biosensing, and neuromorphic computing.^7–14^

The main drawback of self-assembled QDs is their random nucleation due to the total energy minimization process during the surface growth, and the difficulty of achieving control over the nucleation density.^15,16^ In order to control nucleation of QDs, several methods have been developed, including surface patterning techniques, such as tetrahedral recesses, nanohole patterning^17–24^ or the buried stressor method.^25^ In this work, we present a detailed optical investigation of stressor apertures and positioned QDs, offering a deeper understanding of growing site-controlled quantum dot (SCQD) via the buried stressor method. This includes an analysis of the correlation between the stressor aperture width and the emission and nucleation properties of the QDs. Furthermore, we demonstrate monolithic growth of positioned QDs via the buried stressor method, where we show SCQD nucleation with local control over the QD density via the oxide aperture size on the same chip. This provides a platform for the scalable fabrication of devices, in which low- and high-density QD growth can be realized locally. Examples of this are the emitter array of quantum light sources and microlasers that are grown within a single epitaxial growth of the active layer.

## Results and Discussion

### Sample Growth and Fabrication

The sample was fabricated by two-step epitaxial growth in a horizontal Aixtron 200/4 MOCVD reactor on a GaAs (001) substrate, using the following group III and group V precursors: trimethylgallium (TMGa), trimethylindium (TMIn), trimethylaluminum (TMAl), arsine (AsH_3_), and *tert*-butylarsine (TBA) with hydrogen (H_2_) as the carrier gas. The growth rates and compositions of the binary and ternary compounds were calibrated from high-resolution X-ray diffraction (HRXD) scans of InGaAs/GaAs, AlGaAs/GaAs, AlAs/GaAs superlattices with a Malvern X‘Pert^3^ Material Research Diffractometer and from the in situ reflectance anisotropy spectroscopy measurements via a LayTec EpiRAS system.

In the first growth step, a 200 nm GaAs buffer layer is grown after an initial oxide desorption process in an AsH_3_-rich atmosphere, followed by the deposition of 33.5 distributed Bragg reflector (DBR) mirror pairs, consisting of 78.5 nm Al_0.9_Ga_0.1_As and 66.8 nm GaAs layers. Subsequently, a stressor layer stack is grown, comprising a 10 nm Al_0.1→0.9_Ga_0.9→0.1_As graded layer, a 40 nm Al_0.9_Ga_0.1_As layer, a 30 nm AlAs layer, a 40 nm Al_0.9_Ga_0.1_As layer, and a 10 nm Al_0.9→0.1_Ga_0.1→0.9_As graded layer. Finally, a 80 nm GaAs layer is deposited. The temperature at which all layers are grown in this step is 700 ^◦^C.

The sample is then removed from the reactor and processed in a clean room. First, a 0.5 μm-thick layer of AZ nLOF 2070 photoresist is spin-coated onto the sample at 4000 rpm.-grade. Subsequently, the sample is baked at 110 ^◦^C for 60 s. This is followed by the electron beam lithography (EBL) patterning of square mesas with various sizes in the range of 20.3–24.9 μm with a step size of 0.1 μm. After exposure, the sample is baked again at 110 ^◦^C for 60 s and developed in AZ 726 MIF developer for 15 s. After development, the sample is etched anisotropically using a Sentech SI 500 inductively coupled plasma reactive ion etching (ICP-RIE) system to uncover the buried AlAs layer, after which it is cleaned in a 70 ^◦^C dimethyl sulfoxide (DMSO) solution. Further details of the etching process can be found in the Supporting Information (SI), section S6. The initial growth and fabrication steps are schematically represented in Figure 1a. After any residuals are removed, the sample is laterally oxidized in an in situ oven with water vapor as the oxidizing agent and nitrogen (N_2_) as the carrier gas (Figure 1b). This step is similar to the lateral oxidation step in vertical-cavity surface-emitting laser (VCSEL) processing.^26^ However, here the oxidized layer serves not to confine current in the active region, but to modify the surface strain profile at the QD growth surface in order to induce site-controlled growth. A cross-section of the resulting strain distribution can be found in Gaur et al.^27^ The oxidization is performed at 405 ^◦^C; as for the temperatures closer to 390 ^◦^C, lower oxidation speed anisotropy between the ⟨100⟩ and ⟨110⟩ directions is present for similar oxidation conditions.^28^ This ensures higher symmetry of the oxide apertures, especially for the smaller aperture sizes. The process is stopped once the smallest patterned mesas (20 μm) are fully oxidized, as can be observed via an optical microscope. The aperture sizes are then defined by the step size (0.1 μm) between the mesa sizes. The total oxidation time was 15 min. The remaining larger mesas then accommodate oxide apertures of different sizes, which determine the behavior of the SCQD nucleation. In Figure 1c, we show examples of a mesa with smaller (bottom) and larger (top) size, measured with a confocal laser scanning microscope (CLSM). The width of the oxide apertures can be obtained from these images via fitting with a step function, as explained in detail in the SI Section S2. When discussing the oxide aperture dimensions, we refer to the widths obtained via this method. The difference in aperture size between the smaller (d_1_) and larger (d_2_) mesas can be seen. The variety of mesa sizes, and therefore the oxide aperture sizes, creates different surface strain profiles above the stressor apertures.^29^ The oxidized sample is then cleaned in deionized water prior to the second growth step.

**Figure 1:**
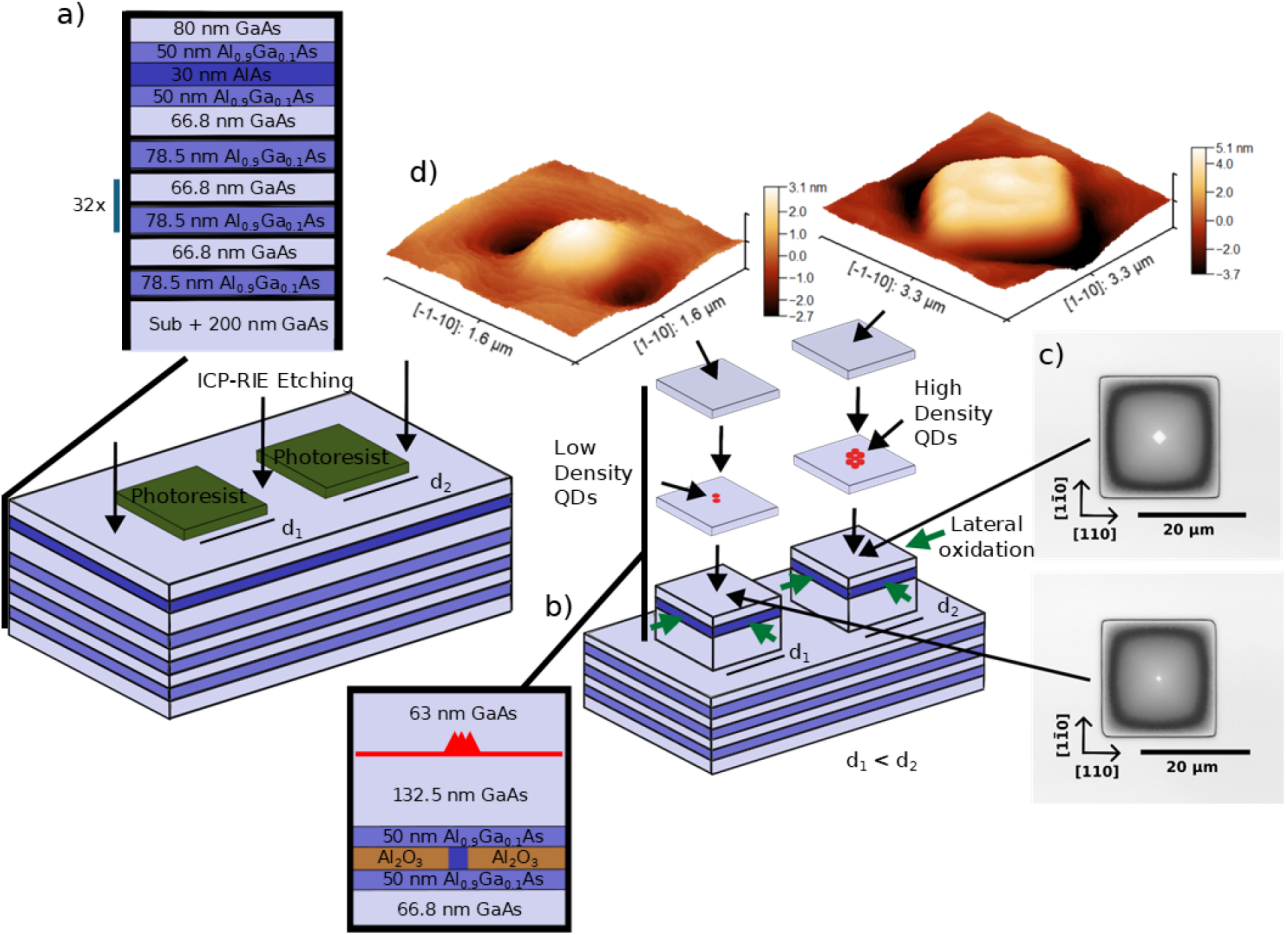
Schematics of the sample fabrication. (a) Layer design and illustration of the ICP-RIE step to uncover the AlAs layer. (b) Illustration of the in situ lateral oxidation of the AlAs layer which results in a change of the surface strain profile. (c) CLSM images showing oxide apertures, where the different aperture sizes can be observed for smaller (bottom) and larger (top) mesa. (d) Self-aligned defect which can be resolved on the surface via AFM.

In the second growth step, a 52.5 nm-thick GaAs layer is grown at 700 ^◦^C, after oxide desorption. The reactor temperature is then decreased to 500 °C, followed by the growth of 2.7 monolayers (ML) of In_0.52_Ga_0.48_As with the growth rate of 0.2 ML/s. The QD layer thickness is above the critical thickness for Stranski-Krastanov nucleation, and the V/III ratio during the active layer growth was 1.5. The combination of the deposit’s low thickness, lower growth rate and lower V/III ratio results in very low planar QD densities.^30–32^ As a consequence, very high selectivity for the QD nucleation above the stressor aperture can be achieved. The deposition is followed by a 50 s growth interruption time with no flux of the V and III precursors. The QDs are then overgrown with a nominally 1 nm thick GaAs capping layer. After capping, an In desorption step is performed by increasing the reactor temperature to 615 °C for 240 s. This step is introduced with the intent to achieve higher QD uniformity. Once the desorption step is completed, the final 63 nm GaAs layer is grown at that temperature. In Figure 1d, atomic force microscopy (AFM) surface profiles after the growth of the final GaAs layer are shown for smaller (left) and larger (right) mesas. We observe the formation of a self-aligned defect above the QDs, as reported by Shih et al.^58^ Different morphologies of the defect can be resolved for both smaller (left) and larger (right) mesas. This distinction is a result of the individual underlying surface strain profile.

### Experimental and Theoretical Results

In order to perform a systematic, quantitative study of the effect of oxide apertures on QD nucleation, it is necessary to determine the relationship between the mesa size and the oxide aperture size, as well as the distribution of oxide aperture sizes on the wafer. For this purpose, a reference sample was grown and processed under conditions that were identical in all respects, with the exception of a reduced number of bottom DBR mirror pairs. As shown in the work of Gaur et al.^27^ the surface strain is influenced by the thickness of the AlAs stressor layer and the thickness of the deposited layers between the QD surface and the stressor. The layers below the stressor are not expected to contribute to the surface strain profile. Similarly, since the aperture sizes are given by the lateral oxidation of the AlAs layer, no DBR-dependent changes in the oxidation properties are to be expected if the fabrication conditions are kept the same.

In Figure 2a, the evolution of the oxide aperture size with the mesa size is shown. To demonstrate the differences caused by fabrication, we present a comparison of the aperture evolution at two separate representative positions on the wafer, i.e., the center (midpoint of the sample) and the edge (near the sample boundary), respectively. The sample studied was a 1/4 piece of a 2 in. wafer, the distance between the measured fields was approximately 1 cm. The two data sets represent the mean aperture sizes of 4 × 4 patterned fields, corresponding to a selected area of roughly 110 mm^2^ on the wafer. The aperture sizes were investigated using CLSM. First, images of mesas with various sizes were acquired and the aperture widths were determined by fitting of the aperture region with a step function. Furthermore, the center of the aperture is determined as the central point of the fitted step function, offset from the actual mesa center. The center of the mesa is found relative to the mesa border through edge detection in the CLSM image. These procedures are explained in further detail in the SI, section S2. The difference in the aperture size between the center and the edge positions of the individual mesas in Figure 2a is due to the inhomogeneous oxidation in the in situ oven. This results in larger oxide apertures in smaller mesas at the sample edge compared to those at the center. Other aspects, such as thickness inhomogeneity from template growth and photoresist thickness inhomogeneity from spin-coating, also influence the outcome of the fabrication process. In both cases, there is a clear linear dependence for aperture sizes of 500 nm, with slopes of 1.08(2) and 1.07(1) for the center and edge pieces, respectively. These values are consistent with each other within the uncertainty, reflecting the robustness of the oxidation process. It is noteworthy that, despite the variation in aperture size across the wafer, the positioning of the apertures relative to the mesa center remains unchanged, as can be seen in the histograms of the lateral and vertical offsets of the measured apertures at the edge and center of the sample. The respective histograms are shown in Figure 2c and Figure 2d. The points and error bars in Figure 2b represent the mean value of the measured aperture offset from the center of mesa for a given mesa size, along with its standard deviation, respectively. As illustrated, the absolute lateral displacement of the oxide aperture remains below 60 nm for the individual mesas with an average value of $17_{-17}^{+19}\mathrm{nm}$ .

**Figure 2:**
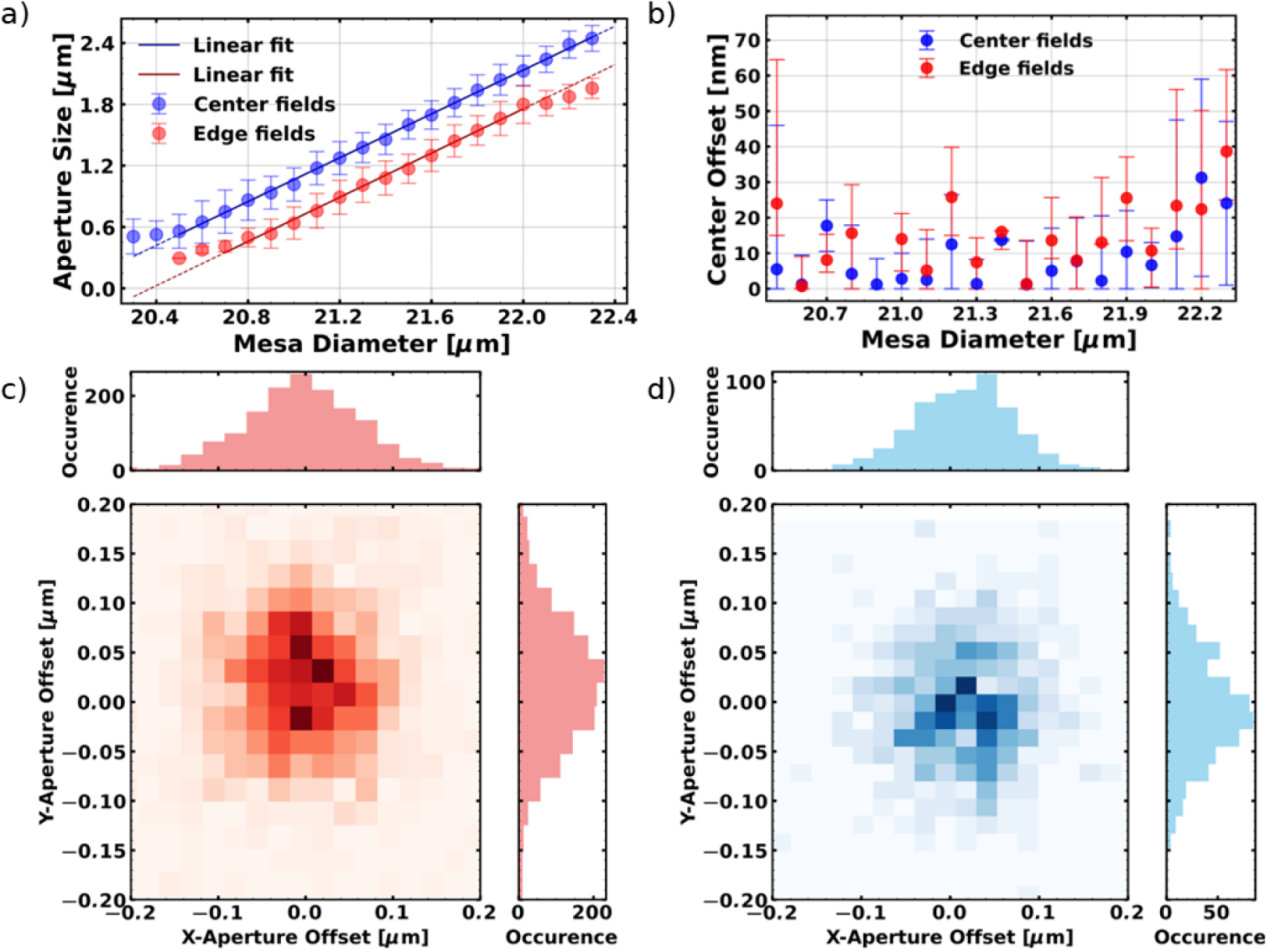
CLSM investigation of stressor apertures. (a) Mean aperture sizes for individual mesa sizes are fitted with linear functions. (b) Absolute aperture offset from the mesa center showing very small aperture displacements. Panels (c) and (d) show the histograms for aperture offsets on sample edge and center, respectively.

To better understand the nucleation behavior and emission properties of SCQDs, we conducted extensive cathodoluminescence (CL) and microphotoluminescence (μ–PL) investigations. First, we focused on the statistical evaluation of the emission properties obtained from μ–PL measurements. Similar to the aperture investigation, we have selected neighboring fields on two positions on the wafer.

From the measured spectra, we can obtain the mean QD emission. This is defined as the mean wavelength of all the peaks identified in the spectra taken for mesas of a given size. These spectra are also used to calculate the mean number of QD peaks. The integrated emission is obtained by integrating the area under the emission spectrum within the range of 905–960 nm using Python’s built-in integrating functions. The peak detection procedure, the emission area integration and the evaluation are discussed in detail in the SI, section S4. Python’s built-in find_peaks­() function is used to detect the lines in each spectrum, which are then fitted with a Lorentzian. Thus, parameters for each measured mesa size are extracted, and a clear trend emerges in both the wavelengths of QD emission, and the observed number of emission lines. We assume mixtures of neutral excitons (X), positive (X^+^) and negatively charged excitons (X^–^). Biexcitons and excitonic complexes with higher number of carriers can be neglected because of the low excitation power, well below the saturation of X, X^+^, and X^–^. The statistical evaluation in Figure 3a incorporates lines of all the mentioned excitonic complexes. We assume a correlation between the number of QDs and the appearance of X, X^–^, and X^+^ lines. The details can be found in Section S4 of the SI. The dependence of the extracted quantities on the mesa sizes is shown in Figure 3a–c, respectively. In Figure 3a, we observe a discrepancy between the largest shift of the emission wavelength between the center (20.7 μm) and edge (21.1 μm) positions. We attribute this to the inhomogeneity of the oxide apertures induced by the sample fabrication, namely by the oxidation process. This results in the aperture size in the center of the sample being larger than on the edge for equivalent mesa sizes. As such, smaller apertures on the center (edge) will be present in smaller (larger) mesas. A qualitative comparison can be made with the CLSM measurements of the aperture sizes, shown in Figure 2a. This also gives rise to the difference between the maxima of the integrated emission area (Figure 3b) and the maximum of QD peaks (Figure 3c), which can be observed for mesa sizes around 21.1 and 21.4 μm for sample center and sample edge, respectively. Here, these extrema are used to indicate the highest SCQD density. In SI, section S4, we present the mean QD emission wavelength, the mean integrated QD emission area, and the mean number of QD peaks shown in Figure 3a–c also as a function of aperture sizes, based on the CLSM measurements.

**Figure 3:**
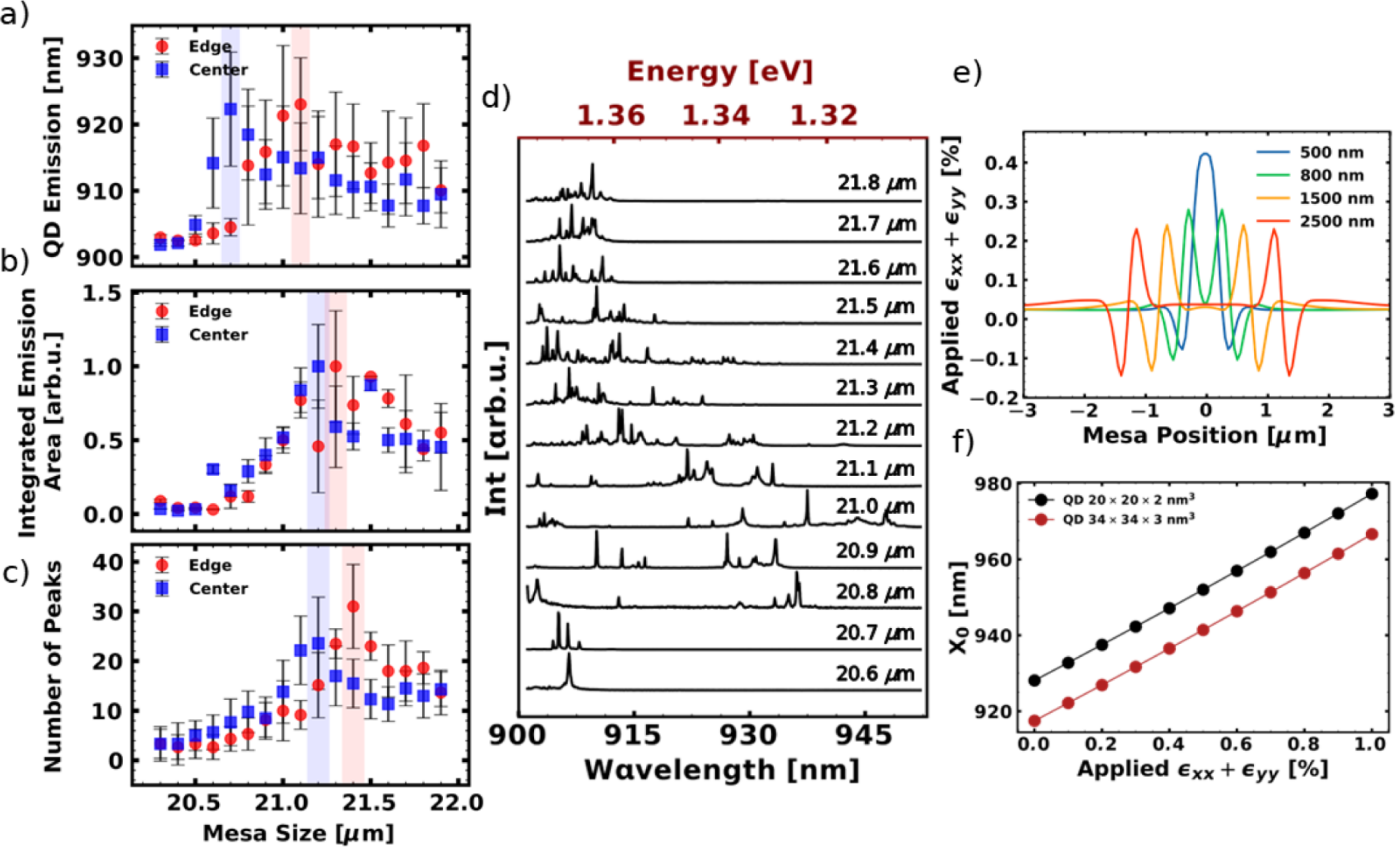
μ–PL investigation of the QD emission and theoretical calculations of the biaxial strain. Panels (a–c) depict the mean QD emission wavelength, the mean integrated QD emission area, and the mean number of QD peaks, respectively. The highlighted regions show the highestmaximum values. (d) Selected spectra for various mesa sizes from a single patterned edge field. (e) Continuum elasticity theory calculation of the biaxial surface strain profile for different aperture sizes. (f) Calculated exciton wavelength shift with respect to the applied biaxial strain. All QD spectra were excited with a tunable pulsed laser at 890 nm. The excitation power was kept constant at 1.5 μW.

Since the surface strain profile exhibits a significant unimodal maximum for smaller aperture sizes of around 500 nm (see Figure 3e), the SCQDs experience the largest applied strain. As can be seen from the calculated wavelengths in Figure 3f, the emission shift between the unstrained dots (ϵ_*xx*_ + ϵ_*yy*_ = 0.0%) and the dots experiencing strain from a 500 nm aperture (ϵ_*xx*_ + ϵ_*yy*_ = 0.4%) corresponds to roughly 20 nm. These results are obtained using continuum elasticity and eight-band **k**·**p** calculations with Coulomb interaction multiparticle correction obtained using the configuration interaction (CI) method, shown in Figure 3e and f. Details on the numerical calculations can be found in the SI, section S1. The QD dimensions in the simulations correspond both in terms of morphology and composition to typical planar Stranski-Krastanov InGaAs QDs.^33^ The highest mean QD wavelengths displayed in Figure 3a demonstrate a comparable wavelength shift from the unstrained QD lines as is suggested by the theoretical results. Consequently, for these mesa sizes, the aperture size at the edge and at the center positions of the sample must be approximately 500 nm.

In Figure 3e, we see that with increasing aperture width, the strain profile undergoes a change from unimodal to bimodal distribution. At the same time, the magnitude of tensile strain decreases from 0.4% to 0.2% as the aperture width increases from 500 to 2500 nm. This decrease in the applied strain with increasing aperture width results in a reduction of the wavelength shift of the QD emission, as can be seen in Figure 3f. On the other hand, the increase in aperture width provides a substantially larger area for QDs to nucleate. We find that the optimum for the mesa size difference between the low and high QD densities is approximately 0.4–0.5 μm. This value is based on the difference between the maximum of the mean QD emission wavelength (Figure 3a), indicating smaller apertures and lower QD densities, and the maxima of the integrated emission area and the number of peaks, exhibiting the highest QD density (Figure 3b and c). In Figure 3d, we show selected spectra for several mesa sizes from a single patterned field on the edge piece of the wafer. The measurements show a noticeable wavelength shift of the QD lines in the low-density spectra for a mesa size of 21.0 μm (corresponding to the aperture size of 500 nm), caused by the applied biaxial strain. The number of emission lines then increases, reaching a maximum at a mesa size of 21.4 μm (corresponding to an aperture size of 890 nm). This is accompanied by a gradual decrease in the QD emission wavelength and the number of QDs.

Additionally, we performed polarization-dependent measurements to explore whether the size of the mesa, and thus the surface strain induced by the oxide aperture, affects the fine structure splitting (FSS) of the nucleated SCQDs. An example of such a measurement can be found in the Supporting Information (SI), section S4. It is anticipated that the FSS will change in response to any distortion in the symmetry of the dot induced by higher values of anisotropic in-plane strain from the stressor aperture.^34^ For an isotropic in-plane strain distribution, as assumed in our theoretical calculations, the FSS values are expected to remain unchanged.^35^ Figure 4a and b illustrate the measured FSS values for different aperture sizes and the theoretical excitonic calculations of FSS dependency on the applied biaxial strain using the CI method.

**Figure 4:**
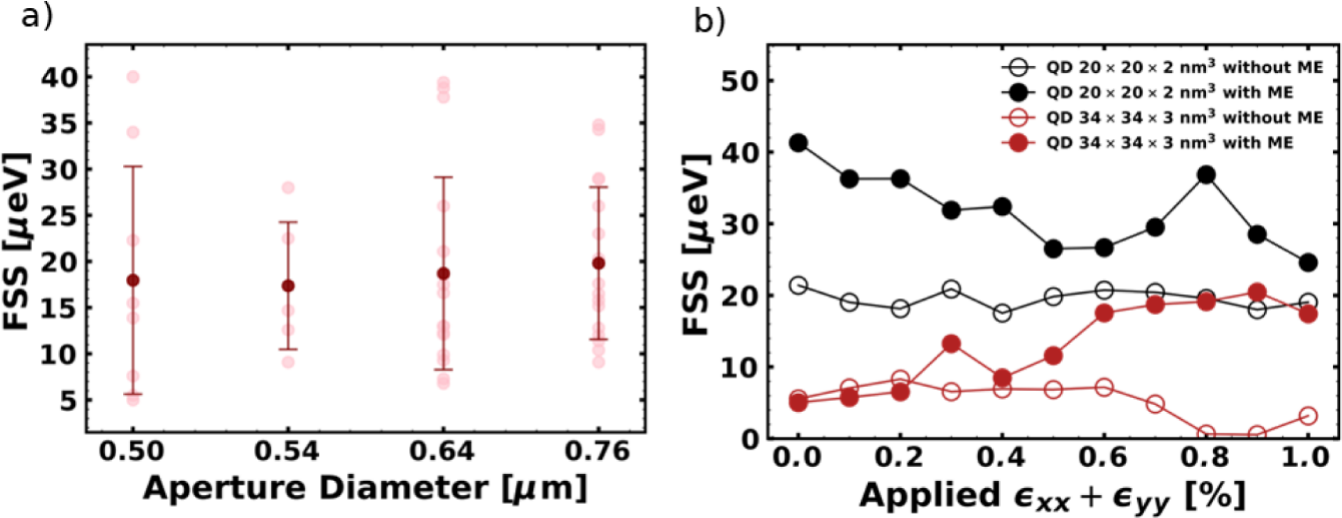
A comparison of measured and simulated FSS. (a) FSS dependency on the aperture size, measured from low-density spectra of the SCQDs. (b) Calculated FSS values for QDs with dimensions of 20 × 20 × 2 nm^3^ and 34 × 34 × 3 nm^3^. The abbreviation ME in (b) refers to the use of the multipole expansion of the exchange interaction in our calculations.^36,37^ The slight cusps and dips in the dependencies in (b) are due to numerical errors in the calculations.

The mean value of the measured FSS of the low-density lines for the aperture sizes of 0.50–0.76 μm (corresponding to mesa sizes of 20.7–21.0 μm) is 19.5(9.6) μeV. The aperture sizes are based on the CLSM measurements. This value is comparable to those observed in self-assembled InAs/GaAs QD samples grown on a substrate with the same orientation.^38–40^ However, it is significantly larger than the homogeneous emission line width in the range of 1 μeV, meaning that temporal postselection or postgrowth treatment is needed to generate polarization-entangled photon pairs using the biexciton–exciton cascade.^41–44^ As can be seen in Figure 4a, there is almost no change in the FSS splitting within this range. Therefore, we conclude that the applied biaxial strain has little to no effect on the QD or the substrate anisotropy. This conclusion is reinforced by theoretical excitonic calculations using the CI method, which show similar FSS values as those observed in the experiment (see Figure 4b). Here, we show FSS calculations for two typical truncated-cone-shaped InGaAs QD sizes, i.e., one with a basis of 20 nm and a height of 2 nm and the other with a 34 nm basis and a 3 nm height. Moreover, for each aforementioned QD structures in Figure 4b, FSS for the case when the multipole expansion of the exchange interaction^36,37^ was (was not) considered in CI is shown by full (open) symbols. The principle of multipole interaction is discussed in detail in works of T. Takagahara^36^ and Křápek et al.^37^ The method provides correction to Coulomb interaction calculations in InGaAs QDs by expansion of Kane’s parameter. We can deduce from Figure 4b that (i) the smaller dot (20 nm base and 2 nm height) shows larger FSS magnitudes than the larger dot (34 nm base and 3 nm height) and (ii) incorporation of multipole expansion of exchange increases FSS even further for each dot size.^45^ Noticeably, while for the case without multipole expansion the FSS does not change appreciably with increasing tensile biaxial strain, when that is switched on a slight overall change of the FSS with applied strain is observed. Interestingly, in the latter case, the FSS increases (decreases) with applied biaxial strain in the case for the larger (smaller) dot. Nevertheless, all aforementioned computed magnitudes of FSS lie within the error bars of the experimentally observed values in Figure 4a. Our results indicate an isotropic in-plane strain distribution of the smaller oxide apertures below 0.8 μm by showing no change of the FSS magnitude with increasing aperture size. Considering QD nucleation, the QDs are highly similar with respect to their morphology and composition. For larger aperture sizes, with square-like arrangement of QDs (as can be seen later in Figure 5c), the aperture and the strain becomes in-plane anisotropic, with 4-fold symmetry (C_4_), as is shown in the Supporting Information of ref. 29. Here, it is impossible to perform such an FSS analysis due to the presence of an increased number of QD lines. In order to measure the strain distribution of the apertures in detail, high-resolution techniques, such as X-ray nanodiffraction, need to be implemented.^46,47^

**Figure 5:**
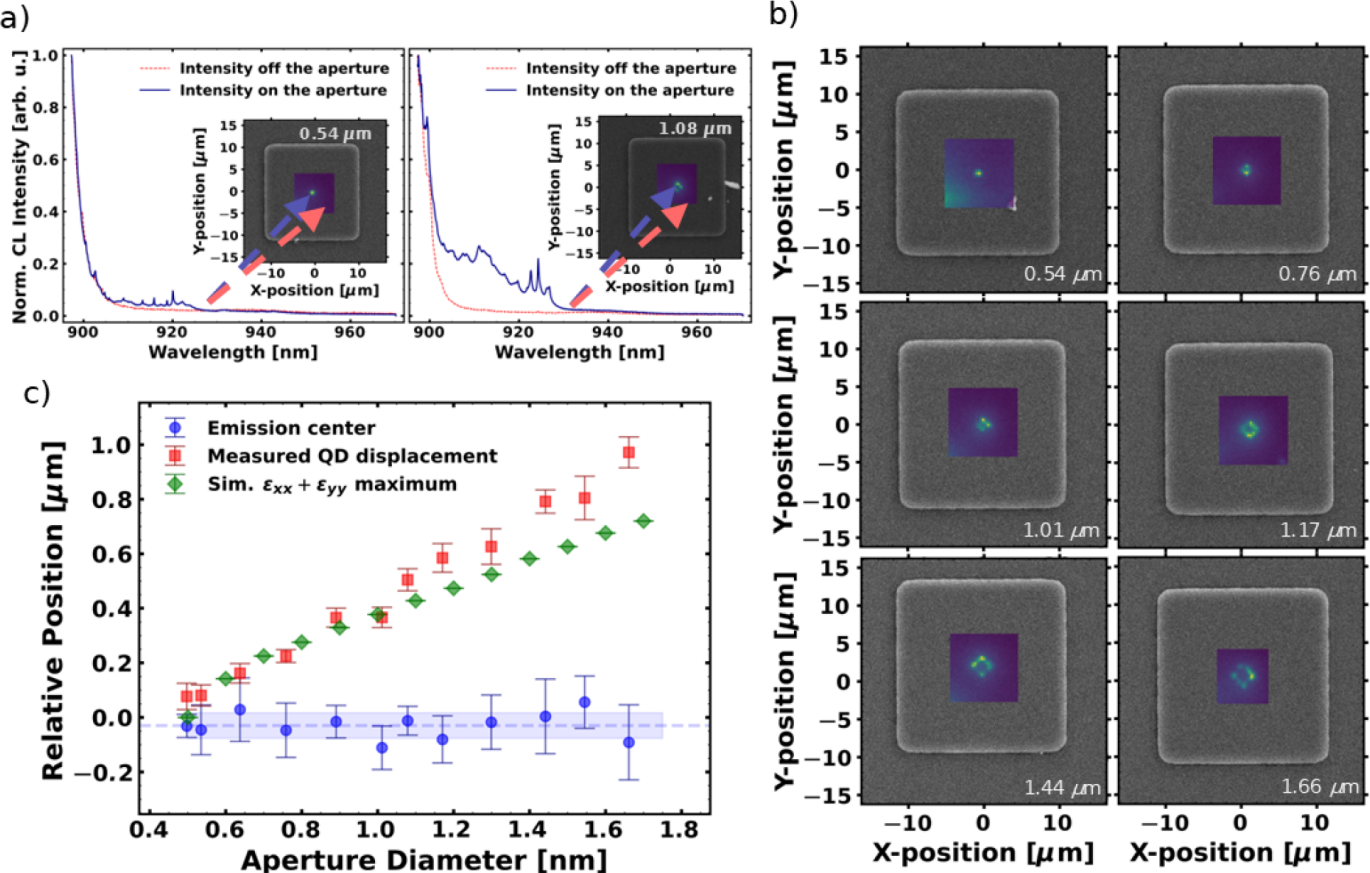
SCQD investigation via CL mapping. (a) Selected CL spectraintensity profiles taken on and off the aperture. The insets show the corresponding SEM images overlaid with the respective CL maps, for aperture sizes of 0.54 μm (20.8 μm mesa) and 1.08 μm (21.3 μm mesa). (b) SEM images overlaid with their respective CL maps for increasing aperture sizes. (c) Dependency of the CL-measured QD displacement (red), CL-measured emission center (blue), and the simulated positions of the tensile strain maxima (green) for increasing aperture sizes.

Figure 5a, shows exemplary low- and high-density CL spectra for a selected mesa with smaller (0.54 μm) and larger (1.08 μm) aperture size. These aperture sizes are based on CLSM measurements. The insets depict the corresponding SEM images overlaid with the acquired CL maps. The maps and the spectra show that the emission is centered in the middle of the mesa. As we increase the aperture size, we observe a change in the distribution of the nucleated QDs, as can be seen in Figure 5b. This can be attributed to the transition from the unimodal to the bimodal strain distribution. As the aperture becomes wider, the QDs are positioned at its edges, specifically at the positions of maximal tensile strain. This preferential nucleation of dots at the positions of the tensile strain maximum is caused by the reduced lattice mismatch between the deposit and the substrate at these positions. This results in a local decrease in free energy for QD growth, favoring QD nucleation at the positions of the tensile strain maximum. Consequently, a transition from a point-like arrangement of QDs in the smaller apertures to a square-like arrangement in the larger ones can be observed, with the arrangement becoming increasingly uneven as the aperture size increases.

The QD displacement and emission centers are calculated using fits to the radial cuts of the intensity distribution. The mesa center is defined as the center of the square formed by the intersections of lines parallel to its edges. Radial cuts of length 5 μm are extracted at 10° intervals across the mesa center to obtain the intensity profiles, which are then individually fitted with a Gaussian or double Gaussian function, based on the shape of the acquired intensity profile. The positions of the maxima obtained from the fit provide the QD displacement from the mesa center as a Euclidean distance. We also consider the maximum of the unimodal distribution to be the emission center. For the bimodal distribution, the emission center is defined as the center of mass between the two maxima. The fitting procedure is described in detail in the SI, section S3.

In Figure 5c, we show the QD displacement and the QD emission offset from the mesa center of one patterned field as a function of the aperture size, depicted as red and blue data-points, respectively. The simulated positions of the tensile strain maxima are shown in green. The QD emission center exhibits a nearly constant value of −30(47) nm. Namely in the case of low-density spectra, such QD displacement has little to no impact on the photon extraction efficiency (PEE) of QDs.^48^ The QD displacement shows dependence on the simulated position of the tensile strain maxima, with strong agreement between the experimental and the theoretical results for smaller apertures of up to 1.17 μm. For larger aperture sizes, we notice a discrepancy between the simulated tensile strain values and the measured QD positioning. This can be attributed to lowered control over the QD positioning due to the lowered tensile strain value and to the anisotropy of the oxide apertures (shown in ref. 29).

In practical applications, QDs are usually coupled to a fabricated resonant cavity, such as a micropillar cavity, a circular Bragg grating (CBG) cavity or a photonic crystal (PhC) cavity.^49–51^ Alternatively, they can be integrated into a waveguide.^52^ In general, any QD displacement can negatively impact the position-dependent light-matter coupling strength of cavity-coupled emitter systems, affecting the Purcell effect and PEE. For example, displacements of more than 50 nm have been shown to have a highly detrimental effect on PEE of CBG, nanobeam, or photonic crystal waveguide cavities.^53^ Furthermore, in the case of CBG cavities, the Purcell factor is also considerably decreased.^54^ On the other hand, the emission properties of QD-micropillars with a larger lateral extent of the confined cavity modes in the range of several 100 nm are less prone to emitter displacements.^55^

Density and position control can be exploited to take advantage of the properties of both high- and low-density QDs simultaneously. This can all be achieved within a single active layer overgrowth. To this end, we created hexagonal arrays involving mesas of two different sizes arranged in an alternating pattern. The size difference between the smaller and larger mesas within each hexagon was set to 0.5 μm. In such a configuration, it is possible to create a device with three reproducible low- and high-density QD nucleation areas for the future realization of SPSs and microlasers, respectively. In this approach, each pair of quantum and classical channels comprises a low-density mesa for quantum key generation and a high-density mesa to provide the communication channel. In this way, it is possible to implement three secure data transmission channels in parallel, effectively increasing the achievable data rate by a factor of 3 when using six-core fibers in a hexagonal configuration, while avoiding crosstalk between the quantum and classical signals.^56^

In order to highlight the uniformity of SCQD nucleation properties within the hexagonal arrays, we present measured hexagons for different aperture sizes in Figure 6. Here, we show μ–PL spectra from hexagonal arrays with aperture sizes of 0.50 and 1.01 μm (mesa sizes of 20.7 and 21.2 μm, respectively) for smaller and larger mesas in Figure 6a, and with aperture sizes of 0.64 and 1.17 μm for smaller and larger mesas (mesa sizes of 20.9 and 21.4 μm, respectively) in Figure 6b. For the case of the hexagonal array with 0.64 and 1.17 μm aperture sizes, we show SEM images overlaid with CL maps in Figure 6c. We observe similar emission characteristics for apertures of the same size. For apertures of sufficiently small sizes (aperture sizes of 0.50 and 0.71 μm), isolated emission lines appear in the μ–PL spectra. In section S5 of the SI, to prove single-photon emission, we present second-order autocorrelation measurements of low-density spectra for a selected hexagon, together with an example of a lifetime measurement.

**Figure 6:**
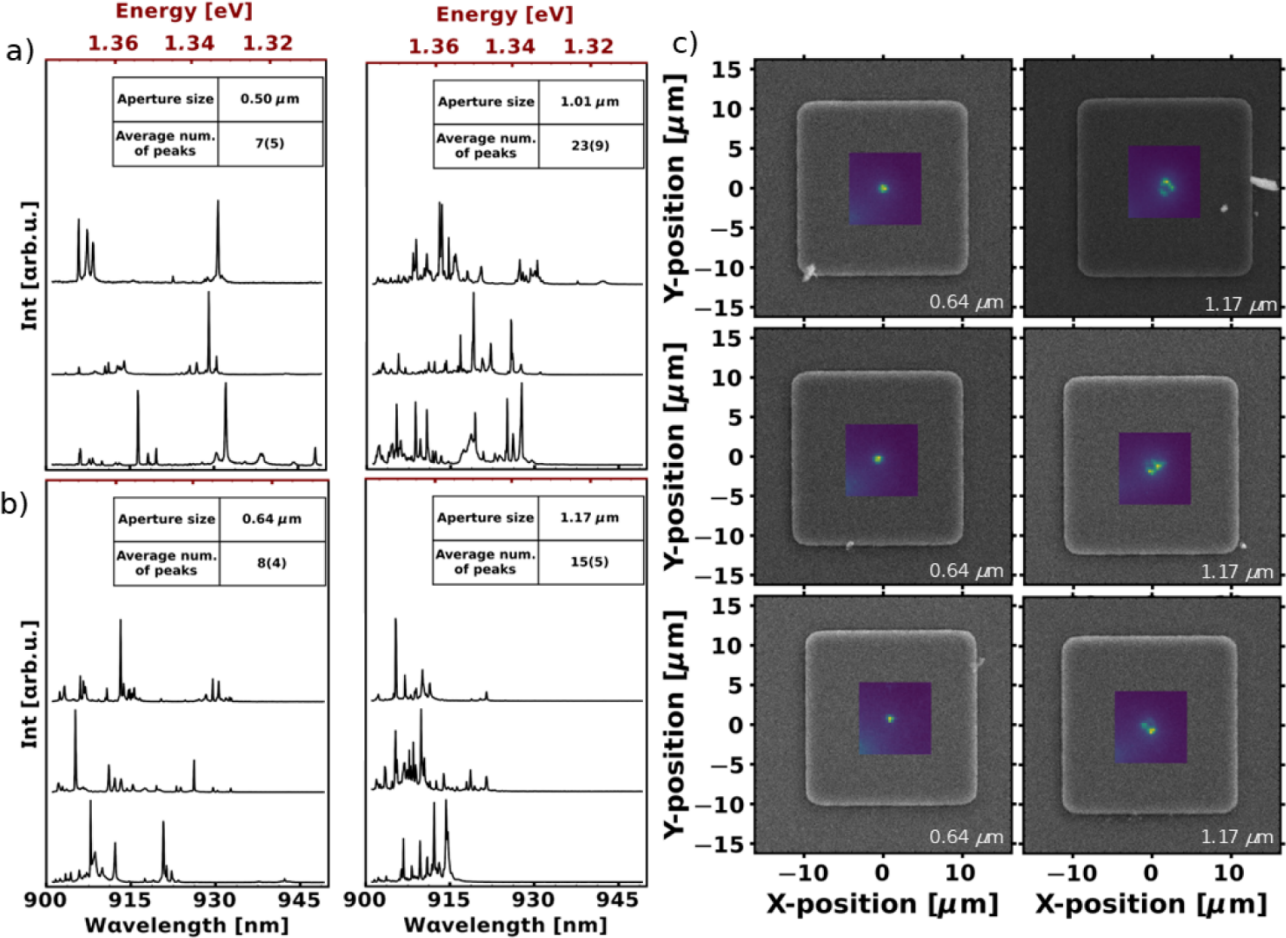
Investigation of hexagonal arrays via μ-PL spectroscopy and CL mapping. (a) QD spectra from a single hexagon with 0.50 and 1.04 μm apertures (20.7 and 21.2 μm mesas, respectively). (b) QD spectra from a single hexagon with 0.71 and 1.35 μm apertures (20.9 and 21.4 μm mesas, respectively). (c) SEM images overlaid with measured CL maps for a hexagonal array, the aperture sizes are identical to (b).

In the CL maps, the emission is always point-like and centered in the middle of the mesa. Here, the tensile strain maximum is unimodal, which allows for the precise nucleation of low-density QDs. For larger aperture sizes (aperture sizes of 1.01 and 1.17 μm), as the strain profile transitions from unimodal to bimodal, the QD spectra exhibit an increased number of lines. The stressor now provides a larger total strained area for QD nucleation. In this instance, the QDs are arranged in a square-like configuration. Consequently, we demonstrate the successful control of QD density via surface strain modulation with the buried stressor method.

Quantum light emission and laser emission of SCQDs grown via buried stressor method have been reported on in previous, separate works.^57,58^ Future work will focus on introducing a suitable resonant cavity design to combine these properties in one sample. If successful, low-output, high-β lasing could be achieved with a limited number of QDs, which has already been reported using nonpositioned QDs by Gies and Reitzenstein.^59^ This would extend the possible applications of SCQDs as an optical gain medium for microlasers into various fields, from coherent excitation sources in the field of quantum nanophotonics,^60–62^ to biosensing, chemical detection, nonlinear optical microscopy and low-power on-chip optical data communication.^63–66^

## Conclusions and Outlook

We have shown a scalable method of site-controlled nucleation of InGaAs QDs via the buried stressor method, with high control over the local nucleation density in emitter arrays. First, we characterized the oxidation process via CLSM. Despite the robust fabrication method, we show high precision of the stressor aperture placement. We achieve a very low lateral displacement of the individual buried stressor apertures of $17_{-17}^{+19}\mathrm{nm}$ . We establish the dependency of the aperture size on the mesa size. Subsequently, we perform statistical μ–PL and CL characterization of the sample, supported by theoretical calculations, explaining the effect of the stressor aperture on the QD emission properties and on the QD nucleation. We show strong agreement between the experimental and the theoretical results. Lastly, we report on the reproducibility of the nucleation properties. We show μ–PL and CL measurements collected for given aperture sizes. These measurements reveal that QD and CL emission spectra are similar for the same aperture size. The results presented in this work provide a deeper understanding of the growth of SCQDs via the buried stressor method, particularly with regard to the correlation between the stressor aperture width and the emission and nucleation properties of QDs. This work lays the groundwork for future applications in the field of quantum photonics, where monolithic devices that combine classical vertical-cavity surface-emitting lasers (VCSELs) and quantum light emitters are integrated onto a single chip. This was achieved by exploiting the controlled growth of QDs with well-defined high and low densities. When paired with a suitable cavity system, such platforms provide a basis for fiber-coupled devices that can enhance fiber-based quantum networks, enabling quantum key distribution (QKD) and quantum repeaters based on entanglement distribution.

## Supplementary Material



## Notes

Principle of the continuum elasticity theory; methodology of the oxide aperture determination with measurement details; figure of the oxide aperture measurement procedure; methodology of the CL measurements with the emission offset determination procedure; figure of the CL emission offset determination procedure; figure of the QD emission offset; methodology of the statistical μ-PL measurements with measurement details; figure of the mean QD emission wavelength, the mean integrated QD emission area, and the mean number of QD peaks with respect to the mesa and the aperture size; details on the time-resolved measurements; figure of power- and polarization-series of a selected SCQD spectrum with the corresponding fits, together with lifetime measurements of X and XX lines; second-order autocorrelation measurements of 3 low-density mesas from a selected hexagonal array; details on the ICP-RIE etching process (PDF)
