# Supplementary material for: Buried Stressor Engineering for Position-Controlled InGaAs Quantum Dots with Local Density Variation for Integrated Quantum Photonics
Source: ACS Photonics. 2026 Jan 7;13(2):471–81. doi: 10.1021/acsphotonics.5c02303 (PMC12828778; doi:10.1021/acsphotonics.5c02303)
Supplement: Supplementary file 1 [file ph5c02303_si_001.pdf]

# Supplementary Information: Buried Stressor Engineering for Position-Controlled InGaAs Quantum Dots with Local Density Variation for Integrated Quantum Photonics

Martin Podhorský,[*,†] Maximilian Klonz,[†] Lux Böhmer,[†] Sebastian Kulig,[†] Chirag C. Palekar,[†] Petr Klenovský,[*,‡,¶] Sven Rodt,[†] and Stephan Reitzenstein[*,†]

†*Institut für Physik und Astronomie, Technische Universität Berlin, Hardenbergstraße 36, D-10623 Berlin, Germany*

‡*Department of Condensed Matter Physics, Faculty of Science, Masaryk University, Kotlářská 267/2, 61137 Brno, Czech Republic*

¶*Czech Metrology Institute, Okružní 31, 63800 Brno, Czech Republic*

E-mail: martin.podhorsky@tu-berlin.de; klenovsky@physics.muni.cz; stephan.reitzenstein@physik.tu-berlin.de

## S1: Continuum elasticity theory

The simulations within this work were performed using the continuum elasticity theory with nextnano++ simulation software.[1] The strain in a solid is described by the dependence of a displacement vector $\mathbf{u}$ on the position denoted by $\mathbf{r}$. The second-rank tensor of infinitesimal strain $\epsilon_{ij}$ is defined as:

$$\epsilon_{ij} = \frac{1}{2}\left\{\frac{\partial u_i}{\partial x_j} + \frac{\partial u_j}{\partial x_i}\right\}. \tag{1}$$

For convenience, the components of the tensor are expressed in terms of particular Cartesian coordinates chosen to agree with the crystallographic axes. The diagonal elements of the tensor $\epsilon_{ij}$ are strain components associated with a change in volume, with its infinitesimal change given by the trace of the deformation tensor. The non-diagonal elements describe the deformation of a volume element, corresponding to a shear distortion. For a homogeneous material, the forces in response to hydrostatic or shear strain are proportional to the area that is affected by the deformation. The stress tensor is symmetric, similarly to the strain tensor. Stresses and strains are related by the Hook's Law and generally the stress tensor can be expressed as:

$$\tau_{kl} = \sum_{ij} c_{klij}\epsilon_{ij} \tag{2}$$

where $c_{klij}$ are the components of the rank-four tensor of the elastic modules. Due to the symmetry of the stress and strain tensors, the following relations for the elastic modules follow: $c_{klij} = c_{lkij} = c_{klji}$. Since the elastic energy is a unique function of the state of strain, the number of the independent components of the elastic tensor is further reduced. The elastic energy density $E_{elast}$ can be expressed as:

$$E_{elast} = \frac{1}{2}\int_V c_{klij}\epsilon_{ij}\epsilon_{kl}dV \tag{3}$$

where $V$ denotes the volume of the whole simulation space. From minimization of $E_{elast}$, we can obtain the desired strain distribution in the simulated structure. It is important to note that this approach lacks the atomistic resolution and for more precise calculations, atomistic theory can be implemented.[2,3] However, the total energy minimization approach still offers adequate precision and has an advantage of lower computational demands when

an appropriate choice of simulation grid is used.

The effect of applied biaxial strain on excitonic energies and FSS was investigated in two types of quantum dots (QDs). First, the effect was examined on a QD with $20 \times 20$ nm base and a 2 nm height. Second, a QD with a base of $34 \times 34$ nm and a 3 nm height was considered. The excitonic energies and FSS were calculated using a combination of the eight-band $\mathbf{k} \cdot \mathbf{p}$ envelope function approximation and Coulomb interaction corrections for electrons and holes, obtained using the configuration interaction (CI) method. A single-particle basis of two-electron and two-hole single-particle ground states was considered. The applied biaxial strain from the stressor aperture was added to the QD strain and the single-particle eigenenergies and eigenfunctions were computed using the eight-band $\mathbf{k} \cdot \mathbf{p}$ and CI.[4] The QD parameters correspond to typical planar Stranski-Krastanov InGaAs QDs in terms of both morphology and composition.[5]

## S2: Oxide Aperture Measurements

The oxide aperture sizes and displacements were investigated using a KEYENCE VK-X 3050 confocal laser scanning microscope (CLSM). A series of automatic measurements mapped parts of the patterned structures over the entire sample surface. The measurements were performed using a 100x magnification objective. The image pixel size is 141.4 nm. The dimension of all measured aperture sizes are above 400 nm and are therefore larger than the pixel size. An example CLSM image of a hexagonal array is shown in Figure S1a, with the oxidation apertures clearly visible as white openings in the center of the mesas. Moreover, the size difference between the openings in the larger and smaller mesas can be observed. As this measurement method allows for fast and precise measurement of oxide apertures, thousands of images were analyzed using an automated mesa detection algorithm to extract the central position of the apertures from the images. Furthermore, the aperture size was measured by taking line profiles of the image intensity across the aperture. The apertures

are square-like in shape, as depicted in Figure S1b. Therefore, three parallel line profiles in close proximity to the aperture, taken along two perpendicular axes, were extracted. These profiles were fitted with a rectangular step function, as shown in Figure S1c. The center of the aperture is defined as the central point of the fitted step function, and the offset to the actual mesa center is calculated. The mesa center is found relative to the mesa border through edge detection in the CLSM image.

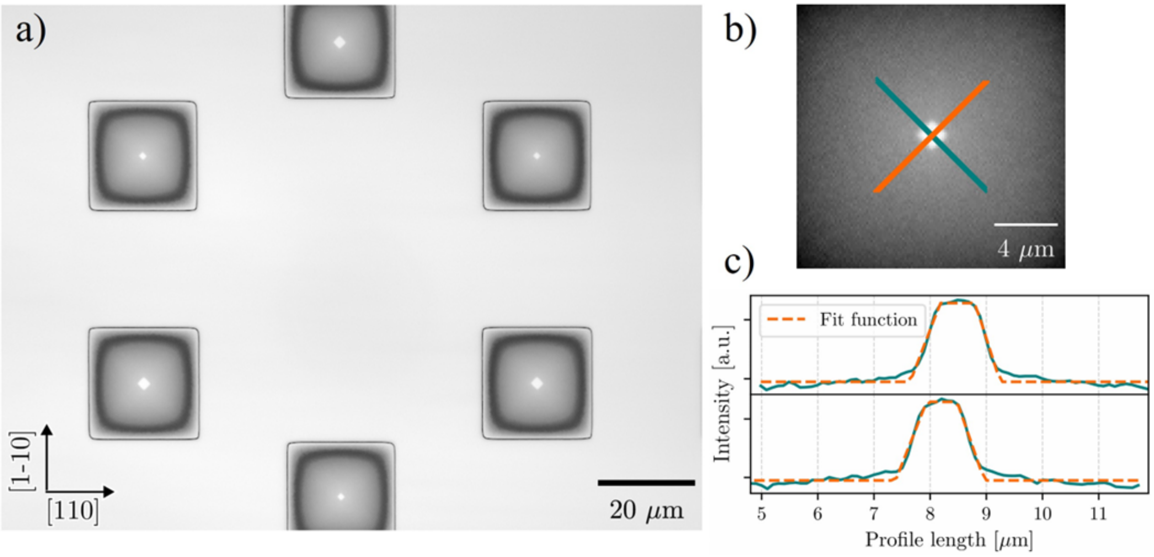


Figure S1: Measurement process of the oxidation apertures. (a) CLSM laser image showing the oxidation apertures as bright openings at the mesa center. (b) Line profiles through the mesa center are used to determine their size and position. (c) Rectangular step functions fitted to the profiles in (b) to extract the aperture dimensions.

# S3: Cathodoluminescence Measurements

The cathodoluminescence (CL) measurements were conducted using a customized Raith eLINE Plus system equipped with a Delmic SPARC CL extension. All CL maps were taken at a temperature of 20 K with an accelerating voltage of 20 kV and a 30 $\mu$m aperture. CL spectra were obtained using a 1200 lines/mm grating and an integration time of 1 ms. The pixel size of the CL maps is 350 nm. The layer structure, especially the $Al_2O_3$ layer regions, result in a very small effective diffusion length. Consequently, effective excitation of

a QD via the electron beam is only possible within a close proximity to the QD of 173(32) nm. This results in quite small and well localized QD emissions spots. The value of the effective excitation length resulting from the combination of direct excitation volume and diffusion contributions of 173(32) nm was calculated from the measured CL intensity profiles for aperture sizes of 0.50–0.64 $\mu$m. The spectral emission window of the CL maps is between 910 and 945 nm. To determine the center of the mesa in the CL maps, we first identified

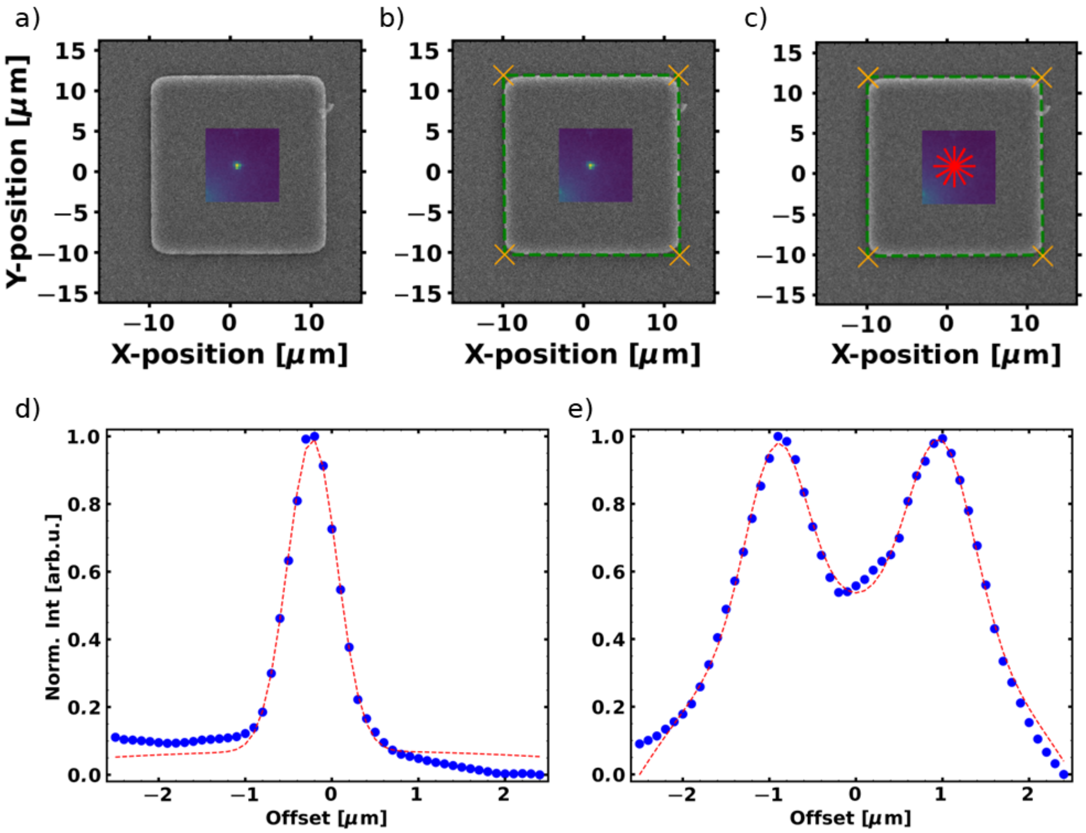


Figure S2: Schematic to illustrate the process of determining the center-point of the CL map. (a) Identification of SEM image overlaid with the measured CL map. (b) Determination of the mesa edges, the intersect of the four points gives the mesa center. (c) Radial cuts across the mesa center. (d) and (e) Gaussian and double Gaussian fits of the unimodal and bimodal intensity profiles, respectively.

the edge of the mesa, indicated by the green lines in Figure S2b. The mesa center is then defined as the center of the square formed by the intersections of the lines. To obtain the QD nucleation position, 18 radial cuts, each 5 $\mu$m long, were extracted at 10° intervals along the mesa center to obtain the intensity profiles, as depicted in Figure S2c. These

are then individually fitted with Gaussian or double Gaussian profiles (see Figure S2d and e). The positions of the fit's maxima give the QD displacement from the mesa center as the Euclidean distance. In the unimodal distribution, the maximum is considered to be the emission center. In the case of a bimodal distribution, the emission center is given as the center of mass between the two maxima.

Figure S3 shows the x- and y-offset of the QD emission center from the mesa center. In case of all aperture sizes, we observe a high positioning accuracy of the nucleated QDs with respect to the mesa center.

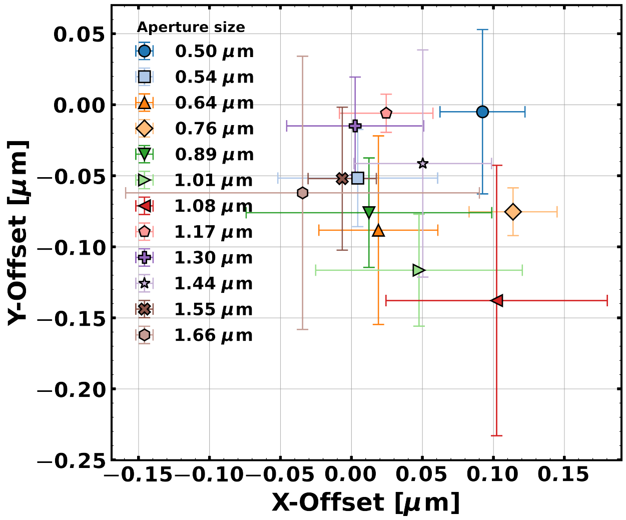


Figure S3: Center of QD emission offset, showing the positioning accuracy of the nucleated SCQDs.

## S4: Micro-Photoluminescence Measurements

The micro-photoluminescence ($\mu$-PL) measurements were conducted at 4 K with the sample mounted in an attoDRY800 closed-cycle cryostat. The QDs were excited with an APE picoEmerald optical parametric oscillator (OPO) tunable pulsed laser set to 890 nm with an excitation power of 1.5 $\mu$W. The laser is directed through a 90:10 beam-splitter. Combined with the losses on the cryostat's windows, this results in approximately 100 nW excitation power on the sample. This low excitation power ensures that only a mixture of neutral

excitons (X), positive ($X^+$) and negatively charged excitons ($X^-$) are present in the spectra. Biexcitons and excitonic complexes with higher number of carriers can be neglected. The QD emission was collected via a LightPath 355330 aspheric lens with 0.77 numerical aperture (NA) and was spectrally filtered with a Teledyne HRS-750 monochromator and detected with a Teledyne PIXIS: 100BR charged coupled device (CCD) camera. The laser signal in the collection path is filtered out with Thorlabs FELH0900 900 nm long-pass filter.

The spectra used for statistical evaluation were measured with a 300 lines/mm grating and a 50 $\mu$m monochromator entrance slit. To obtain the mean emission wavelength, the mean integrated emission area for given mesa sizes and the mean number of QD peaks, we used the SciPy Python libraries for signal processing, integration and curve fitting.[6] First, a threshold value of 50 counts/s is introduced to move above the noise baseline. The peaks in the spectrum are then identified using peak prominence. Prominence is defined as the vertical distance between a peak and its lowest contour line connecting it to a higher peak and is given by the difference between the peak and the surrounding baseline of the signal. The peak prominence of 0.15 of the intensity maximum in the spectrum was used. This makes the peak detection less sensitive to noise. The identified peaks are then fitted with a Lorentzian function. The mean values of the spectral positions of the peaks and the number of identified lines are then taken for each individual mesa size. The integrated emission is obtained by numerically integrating the area under the emission spectrum within the range of 905–960 nm.

In Figure S4a–c, we show the mean QD emission wavelength, the integrated emission area and the number of QD peaks as a function of both the mesa size (left) and the aperture size (right). The aperture sizes are given based on the CLSM measurements. The optimal difference in aperture size for low- and high-density nucleation appears to be near 0.4–0.5 $\mu$m. To conduct the fine structure splitting (FSS) measurements, emission spectra were collected from selected mesas using a 1200 lines/mm grating and a 30 $\mu$m entrance slit. To conduct these measurements, a $\lambda/2$ waveplate and a linear polarizer were introduced into

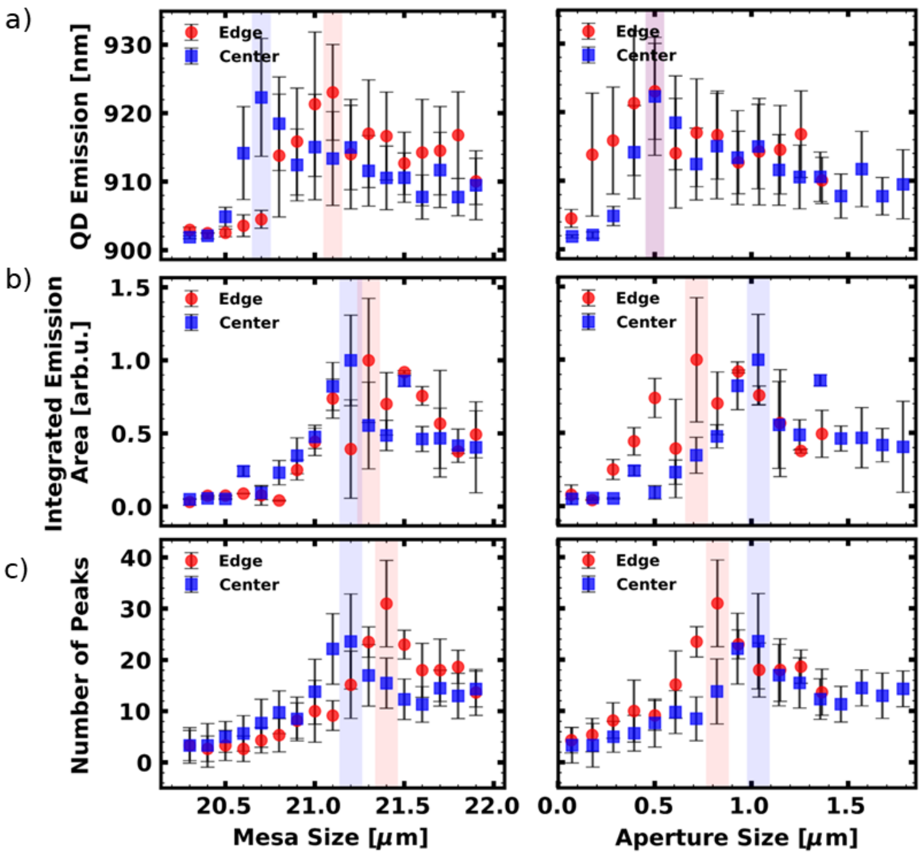


Figure S4: $\mu$–PL investigation of the QD emission. (a–c) show the mean QD emission wavelength, the mean integrated QD emission area, and the mean number of QD peaks with respect to mesa (left) and aperture (right) size, respectively.

the collection path.

## S5: Time-resolved Measurements

The lifetime and the second-order autocorrelation ($g^{(2)}(\tau)$) measurements were performed at 4 K in an attoDRY800 closed-cycle cryostat. The sample was excited using an APE picoEmerald tunable pulsed laser set to 890 nm. The signal was spectrally filtered using a Teledyne HRS-750 monochromator and detected using a Teledyne PIXIS: 100BR CCD camera. A Thorlabs FELH0900 900 nm long-pass filter was used to filter out the laser in the collection path. In Figure S5, we present a polarization- and power-dependent measurement of a selected low-density SCQD spectrum. The individual QD complexes were identified

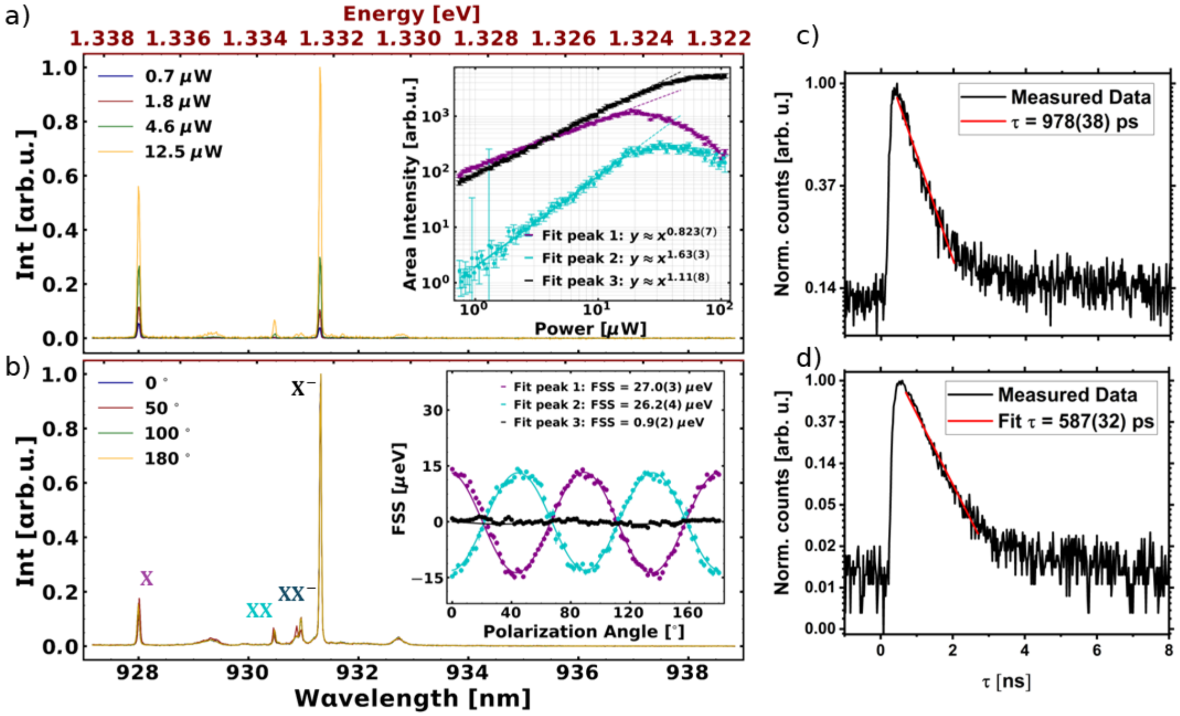


Figure S5: Power- and polarization-dependent measurements of a selected low density SCQD spectrum with corresponding time-resolved measurements. (a) Power-dependent measurement with linear fits of the X (violet), XX (cyan), and $X^-$ (black) lines' power dependencies in double logarithmic scale. (b) Polarization-dependent measurement with sine fits of the X (violet), XX (cyan), and $X^-$ (black) lines' dependencies on the polarization angle. (c) and (d) Lifetime measurements (black) with linear fits (red) of X and XX lines, respectively.

in accordance with refs.[7,8] and are shown in Figure S5b. The lifetime measurements were performed on the exciton (X) and biexciton (XX) lines . The obtained values of 978(38) ps for X and 587(32) ps for XX are comparable to typical self-assembled InGaAs QDs.[9,10] The excitation power for the lifetime measurements was set to 10 % of the measured QD line saturation power ($P_{sat}$). The QD signal is collected via a LightPath 355330 aspheric lens with an NA of 0.77. In Figure S6, we show $g^{(2)}(\tau)$ measurements on low-density mesas of a selected hexagonal array. In all cases, we observe $g^{(2)}(0)$ below 0.5, indicating single-photon generation.[11] For the $g^{(2)}(\tau)$ measurements, the excitation power for each line was set to 75 % of its respective $P_{sat}$. The $g^{(2)}(\tau)$ measurements were conducted with an LightPath 355330 aspheric lens with 0.77 NA to collect the signal.

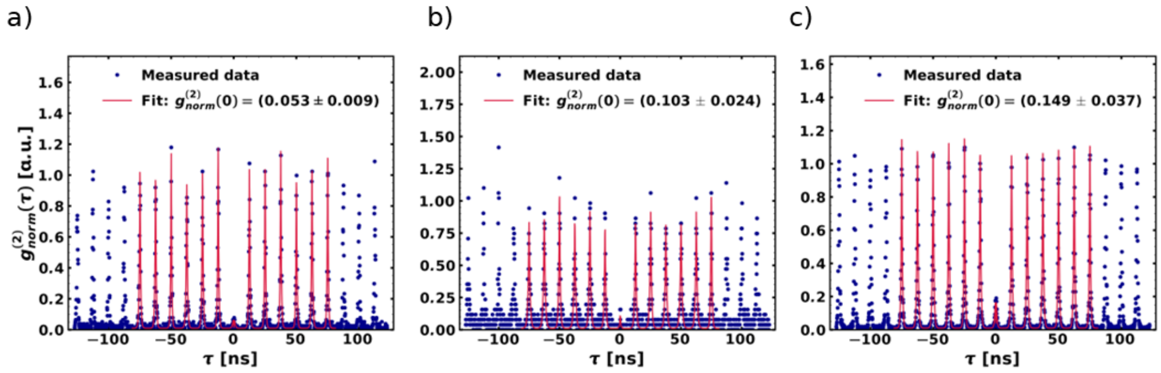


Figure S6: Second-order autocorrelation measurements. (a–c) The second-order autocorrelation measurements of low-density spectra taken from a selected hexagon. In all cases, the $g^{(2)}(0)$ values show single-photon generation.

# S6: Plasma Etching of Samples

The inductively coupled plasma reactive ion etching (ICP-RIE) was carried out using a SI 500 system from Sentech GmbH, Germany. The plasma is controlled via an ICP and a capacitively coupled plasma (CCP) electrode at set powers. The electrode temperature is set to 20 °C. The ICP and CCP powers are 100 W and 40 W, respectively. The bias voltage during the etching is 150–230 V. The reactor pressure was set to 0.08 Pa. The reactive gases are $Cl_2$ and $BCl_3$, as well as Ar for the physical component of the etching process. The flow rates of $Cl_2$, $BCl_3$, and Ar were set to 1.3 sccm, 4.3 sccm, and 1.1 sccm, respectively. The sample is placed on an $Al_2O_3$ chuck with a constant He backside pressure of 1000 Pa.

# References

(1) Birner, S.; Zibold, T.; Andlauer, T.; Kubis, T.; Sabathil, M.; Trellakis, A.; Vogl, P. nextnano: General Purpose 3-D Simulations. *IEEE Transactions on Electron Devices* **2007**, *54*, 2137–2142, DOI: 10.1109/TED.2007.902871.

(2) Sheng, W.; Hawrylak, P. Atomistic theory of electronic and optical properties of InAsInP self-assembled quantum dots on patterned substrates. *Physical Review B* **2005**, *72*, 035326, DOI: 10.1103/PhysRevB.72.035326.

(3) Kratzer, P.; Liu, Q. K. K.; Acosta-Diaz, P.; Manzano, C.; Costantini, G.; Songmuang, R.; Rastelli, A.; Schmidt, O. G.; Kern, K. Shape transition during epitaxial growth of InAs quantum dots on GaAs(001): Theory and experiment. *Physical Review B* **2006**, *73*, 205347, DOI: 10.1103/PhysRevB.73.205347.

(4) Yuan, X.; Covre da Silva, S. F.; Csontosová, D.; Huang, H.; Schimpf, C.; Reindl, M.; Lu, J.; Ni, Z.; Rastelli, A.; Klenovský, P. GaAs quantum dots under quasiuniaxial stress: Experiment and theory. *Physical Review B* **2023**, *107*, 235412, DOI: 10.1103/PhysRevB.107.235412.

(5) Hong, K. J.; Tan, C. H.; Tan, S. T.; Chong, K.-K. In *Graphene, Nanotubes and Quantum Dots-Based Nanotechnology*; Al-Douri, Y., Ed.; Woodhead Publishing Series in Electronic and Optical Materials; Woodhead Publishing, 2022; Chapter 30 - Morphology and topography of quantum dots, pp 727–770, DOI: 10.1016/B978-0-323-85457-3.00009-8.

(6) Virtanen, P. et al. SciPy 1.0: fundamental algorithms for scientific computing in Python. *Nature Methods* **2020**, *17*, 261–272, DOI: 10.1038/s41592-019-0686-2.

(7) Ding, F.; Singh, R.; Plumhof, J. D.; Zander, T.; Křápek, V.; Chen, Y. H.; Benyoucef, M.; Zwiller, V.; Dörr, K.; Bester, G.; Rastelli, A.; Schmidt, O. G. Tuning the Exciton Binding Energies in Single Self-Assembled InGaAs/GaAs Quantum Dots by Piezoelectric-Induced Biaxial Stress. *Physical Review Letters* **2010**, *104*, 067405, DOI: 10.1103/PhysRevLett.104.067405.

(8) Rodt, S.; Schliwa, A.; Pötschke, K.; Guffarth, F.; Bimberg, D. Correlation of structural and few-particle properties of self-organized InAsGaAs quantum dots. *Physical Review B* **2005**, *71*, 155325, DOI: 10.1103/PhysRevB.71.155325.

(9) Xu, Z.; Zhang, Y.; Hvam, J. M. Long luminescence lifetime in self-assembled In-

GaAs/GaAs quantum dots at room temperature. *Applied Physics Letters* **2008**, *93*, 183116, DOI: 10.1063/1.3021018.

(10) Harbord, E.; Spencer, P.; Clarke, E.; Murray, R. Radiative lifetimes in undoped and *p*-doped InAs/GaAs quantum dots. *Physical Review B* **2009**, *80*, 195312, DOI: 10.1103/PhysRevB.80.195312.

(11) Fox, M. In *Quantum Optics: An Introduction*; Fox, M., Ed.; Oxford University Press, 2006; Chapter 6 - Photon Antibunching, pp 105–125, DOI: 10.1093/oso/9780198566724.003.0006.
